# Solid Lipid Nanoparticles (SLN) and Nanostructured Lipid Carriers (NLC) Prepared by Microwave and Ultrasound-Assisted Synthesis: Promising Green Strategies for the Nanoworld

**DOI:** 10.3390/pharmaceutics15051333

**Published:** 2023-04-25

**Authors:** Karla L. López, Andrea Ravasio, José Vicente González-Aramundiz, Flavia C. Zacconi

**Affiliations:** 1 Institute for Biological and Medical Engineering, Schools of Engineering, Medicine and Biological Sciences, Pontificia Universidad Católica de Chile, Santiago 7820436, Chile; 2 Escuela de Química, Facultad de Química y de Farmacia, Pontificia Universidad Católica de Chile, Av. Vicuña Mackenna 4860, Macul, Santiago 7820436, Chile; 3 Escuela de Química y Farmacia, Facultad de Química y de Farmacia, Pontificia Universidad Católica de Chile, Santiago 7820436, Chile; 4 Centro de Investigación en Nanotecnología y Materiales Avanzados, Pontificia, CIEN-UC, Universidad Católica de Chile, Santiago 7820436, Chile; 5 Center for Nanomedicine, Diagnostic & Drug Development (ND3), Universidad de Talca, Talca 3460000, Chile

**Keywords:** nanocarriers, solid lipid nanoparticles, nanostructured lipid carriers, microwave-assisted synthesis, ultrasound-assisted synthesis

## Abstract

Many pharmaceutically active molecules are highly lipophilic, which renders their administration and adsorption in patients extremely challenging. Among the countless strategies to overcome this problem, synthetic nanocarriers have demonstrated superb efficiency as drug delivery systems, since encapsulation can effectively prevent a molecules’ degradation, thus ensuring increased biodistribution. However, metallic and polymeric nanoparticles have been frequently associated with possible cytotoxic side effects. Solid lipid nanoparticles (SLN) and nanostructured lipid carriers (NLC), which are prepared with physiologically inert lipids, therefore emerged as an ideal strategy to bypass toxicities issues and avoid the use of organic solvents in their formulations. Different approaches to preparation, using only moderate amounts of external energy to facilitate a homogeneous formation, have been proposed. Greener synthesis strategies have the potential to provide faster reactions, more efficient nucleation, better particle size distribution, lower polydispersities, and furnish products with higher solubility. Particularly microwave-assisted synthesis (MAS) and ultrasound-assisted synthesis (UAS) have been utilized in the manufacturing of nanocarrier systems. This narrative review addresses the chemical aspects of those synthesis strategies and their positive influence on the characteristics of SLNs and NLCs. Furthermore, we discuss the limitations and future challenges for the manufacturing processes of both types of nanoparticles.

## 1. Introduction

The discovery of new drugs profits from many different scientific disciplines, where the role of computational studies has always played a special role for the deeper understanding of chemical and biological parameters [1,2]. However, even after the careful optimization of their syntheses and activities, a drug’s successful testing in ex vivo or in vivo models is one of the main challenges [3,4,5]. One of the main obstacles in the development of new drugs has always been low solubility in water, impeding efficient absorption of active ingredients, especially when administered orally. In addition to solubility issues, other variables can also intervene with a good absorption of a drug in the human body, especially the low stability in the specific biological environment (e.g., interaction with enzymes), and membrane permeabilities [4,6,7].

Approximately 75% of drug candidates and 40% of pharmaceuticals currently on the market exhibit or have exhibited water solubility issues at some stage of their development, which is especially problematic if one considers the oral and parenteral administration routes as the most common ones [6,8,9].

Currently, there are some known strategies to improve the water solubility of such mentioned pharmaceuticals, for example, spray drying and congealing [10], but they are associated with exposure to high heat or cold respectively, causing evaporation, and the possibility of expulsion of the drug from the matrix, caused by its crystallization in an unstable polymorphic form leading to the formation of disordered chains and/or with undesirable orientation and, consequently, promoting low barrier properties [11]. For this reason, an attractive alternative is proposed, such as the encapsulation of active ingredients in colloidal carrier systems. This type of transporter can be designed to specifically control release kinetics and prevent the degradation of the molecules, thus enhancing biodistribution [1,5,12,13]. Alongside a drastic reduction of side effects often associated with pharmaceutical formulations. The application of drug delivery systems is therefore one of the most powerful tools for the treatment, prevention, and early detection of various diseases [4,5]. However, concerns associated with cytotoxicity and biocompatibility of the components of different colloidal delivery systems have been raised [5,13]. The possibility to efficiently engineer lipid carriers such as liposomes, micelles, nanoemulsions, cubosomes, and other lipid nanoparticles has attracted great interest and resulted in an extremely productive and innovative community [14,15].

The first preparation of nanopellets for oral administration was reported in 1986 [16]. Subsequently, lipospheres formulated as Doxil^®®^ were approved by the FDA in 1995, which stimulated the development of solid lipid nanoparticles (SLN) and nanostructured lipid carriers (NLC), respectively. Later improvements of their formulations were focused on physiological lipids, which show many advantages, such as biocompatibility and degradability, as well as efficient cellular and subcellular targeting [17,18,19,20]. Examples of current advances on this type of carrier can be seen in the treatment of diseases such as cancer and allergies. and even in the development of the COVID-19 vaccines for the efficient delivery of mRNA, showing its growing importance as efficient delivery systems on the pharmaceutical field [21,22]. In the later years, these types of nanocarriers have been produced with several methods, such as homogenization, hot agitation, and solvent displacement, but they tend to be multistep processes, affecting in some cases its physicochemical characteristics, generating bigger size particles and polydisperse formulations, and reduce storage stability [12,19,23,24,25].

Recently, microwave-assisted synthesis (MAS) and ultrasound-assisted synthesis (UAS) stood out as efficient methods for the fabrication of lipid nanovehicles with improved physicochemical characteristics. Especially the size control in the preparation of smaller particles and generally lower polydispersity indices, representing a valuable improvement in the generation of colloidal nanocarriers. This is also potentiated by one of the greater attributes of those synthesized techniques: their green approaches, constituting a novelty in the SLN and NLC formulations, generating vehicles by using higher energy efficiency in less time than previous, classically used methods [24,26,27].

**Figure 1:**
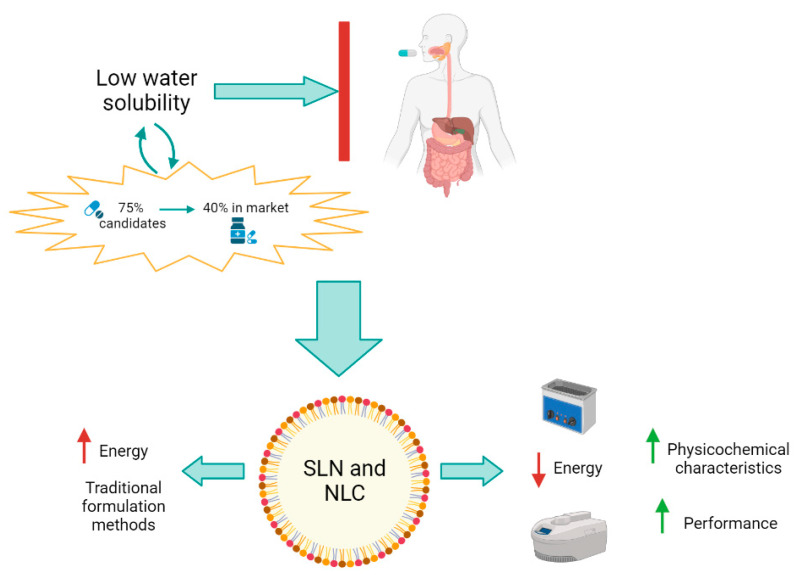
Role of Microwave-assisted synthesis and ultrasound-assisted synthesis as new formulation techniques for the improvement of physicochemical characteristics for SLN and NLC. (Created with BioRender). Red arrows: energy requirement. Green arrows: improvements.

In this narrative review we aim to provide an overview of the state-of-the-art in the microwave-assisted synthesis and ultrasound-assisted synthesis of solid lipid nanoparticles, especially highlighting the advantages of these techniques over conventional protocols, also analyzing their compliance with green chemistry principles (Figure 1). Finally, we will discuss open challenges and will provide a perspective outlook for this type of formulation.

## 2. Solid Lipid Nanoparticles (SLN)

The mobility of the drug incorporated in the colloidal nanocarriers is a parameter of great importance, because if it is high, leaks or release of this drug may occur at an unexpected time or in undesired places [17,28,29]. That is why SLNs offer the encapsulation alternative with less mobility than making formulations with lipid liquids, which is the reason that better physicochemical stability is obtained [17,18].

Solid lipid nanoparticles constitute a novel generation of lipid nanoparticles, prepared by different formulation techniques. The fact that they represent potential drug nanocarriers that overcome side effects presented by other types of known nanocarriers, in addition to their simplicity and versatility, have made them one of the main targets of the current scientific community [17,18,23,28,30,31,32].

At a commercial level, the first product made up of SLN was of a cosmetic nature, coming onto the market in October 2005, under the name Nanorepair Q10^TM^ (by the company Dr Rimpler, Wedemark, Germany) [33]. In the pharmaceutical field, it was in 2018 that they were commercially approved for the first time, as a vehicle for the siRNA drug, Onpattro^®®^. It is then that over time different works have been generated that reflect the efficient incorporation of various drugs in SLN for administration routes, such as parenteral, oral, and transdermal, showing promising results in in vitro studies [15,19,34,35]. Table 1 shows some of the incorporation results corresponding to said research works.

Regarding their structure, SLNs are derived from oil-in-water (O/W) nanoemulsions, where the liquid lipid is replaced by a solid lipid. The elaboration of this type of nanocarriers generally involves temperatures above the melting point of the lipids, followed by cooling so that the liquid droplets crystallize and completely organize, thus replacing them with a rigid solid phase at room or body temperature. This is how the lipid solids can be made up of highly purified triglycerides, complex mixtures of glycerides, or waxes, constituting 0.1 to 30% of the formulation. This solid is stabilized by the use of surfactants (and cosurfactants if necessary) that make up 0.5 to 30% of the formulation and finally the incorporated molecules (make up 5% of the formulation). The rest of the formulation is composed by water [17,23,24,56].

**Figure 2:**
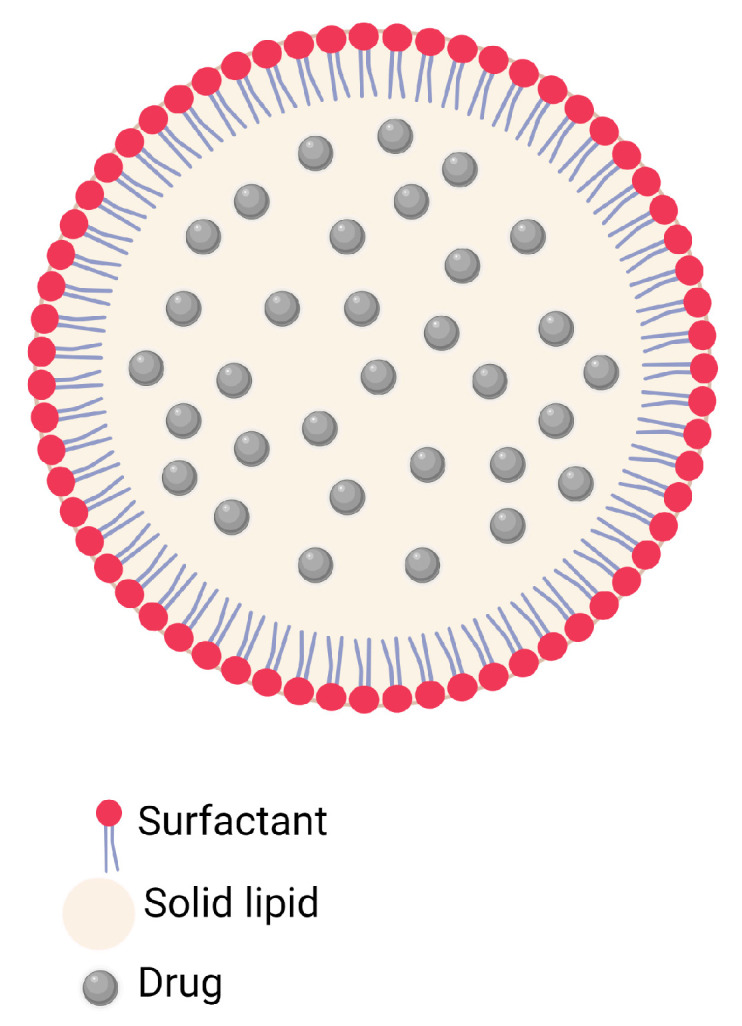
Basic structure of solid lipid nanoparticles. (Created using Biorender).

Figure 2 shows a general schematic representation of the described structure.

The solid core of this type of nanocarrier slows down molecular diffusion and, consequently, the release of incorporated materials, while hindering the entry of pro-oxidants and destructive agents that can internally degrade the internal region of the SLNs before fulfilling their specific function. This class of nanoparticles can be used to incorporate and release hydrophilic and hydrophobic molecules; however, there is a preference towards the incorporation of hydrophobic molecules due to their high affinity with the hydrophobic matrix [19,20,56,57,58].

**Figure 3:**
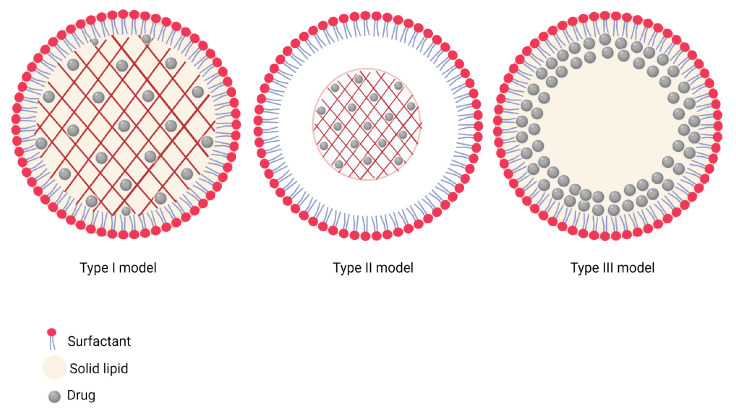
Molecule distribution models in SLNs. (Created using Biorender).

The production conditions, nature, and concentration of excipients have given rise to three possible distribution models of drugs in SLNs observed during different research by transmission electron microscopy (TEM) and scanning electron microscopy (SEM) (schematized in Figure 2) [56,57]. These models (Figure 3) are made up of:

- Type I: a homogeneous matrix distribution, where the controlled release of molecules is favored depending on the dissolution mechanism. The cold homogenization method or hot homogenization method are both appropriate for formulating SLNs of this type, seeking the incorporation of highly lipophilic compounds [56,57].
- Type II: a central model, characterized by a prolonged release of the incorporated components [56,57].
- Type III: an enriched shell when there is a phase separation caused during the cooling of the homogenized nanoemulsion where the first lipid phase participates, gradually increasing the concentration of components in the molten residual lipid with a greater amount of solidified lipid [56,57].

According to their structural characteristics, SLNs are defined by having sizes between 40 to 1000 nm and spherical shapes, also presenting a series of favorable properties that can be listed as [19,24,56]:

- High biodegradability and biostability.
- High capacity for drug absorption and dissolution, improving bioavailability.
- Sustained and controlled drug release.
- Nonuse of organic solvents for their formulations.
- Longest half-life of the drug.
- Longest useful life.

Despite these distinguished benefits offered by SLNs, there are drugs that have greater solubility or compatibility with liquid lipids [6,8]. This can be evidenced at the time of formulating SLN, where applying heat to promote the fusion of the lipid solid facilitates the incorporation of the drug into the matrix. However, when it is dissolved again at room temperature, the crystallization of the lipid (return to the solid state) can generate the expulsion of the drug. That is why the development of new formulations of lipid nanocarriers that could overcome this problem was proposed [28,29,56,59].

## 3. New Generation of Lipid Nanoparticles: Nanostructured Lipid Carriers (NLC)

Nanostructured lipid carriers were first developed in the late 1990s as alternatives to solid lipid nanoparticles and to avoid undesired expulsion of cargo molecules from the matrices [24,60]. NLPs consist of an arrangement in which the nucleus is formed by a mixture of solid lipids and liquid lipids. Generally speaking, solid lipids are characterized by longer chains, as opposed to medium to short chains in liquid lipids or oils, decreasing the overall crystallinity of the nanoformulation as compared to SLNs. These imperfections in the solid matrix the represent small environments to harbor drugs [17,61,62].

Hence the composition of this generation of lipid nanocarriers is based on a blend of solid and liquid lipids, complemented by surfactants (and co-surfactants if necessary) for stability, resulting in particles with sizes ranging from 40 to 1000 nm [17,18,63].

**Figure 4:**
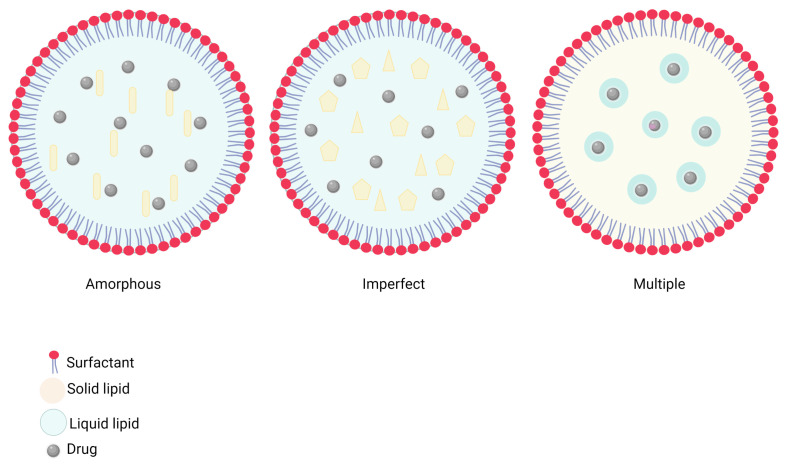
Different types of possible NLC structures. (Created using Biorender).

Studies through TEM and SEM microscopy have shown that NLCs occur mainly in three different structures:

(i) Amorphous type, in which solid lipids form solid particles but do not crystallize perfectly, giving rise to the lipid matrix in a homogeneous amorphous state, greatly reducing the possibility of expulsion of the incorporated molecules.
(ii) An imperfect structure, made up of a disordered matrix with many spaces that accommodate a large amount of active substance in amorphous areas. This decreases the risk of expulsion of the cargo. Nevertheless, the possibility of early expulsion during the transition from the molten to the solid state through lipid crystallization phenomena is still imminent.
(iii) Multiple type structure, resulting from high concentrations of oils, which are well miscible with the molten solid lipid at high temperatures. Upon cooling, the presence of the oil furnishes nanocompartments within the solid lipid matrix. This allows for a favorable better arrangement of incorporated molecules, better encapsulation, enhanced controlled release profiles, and a minimization of premature expulsion of cargo [18,24,60] (Figure 4).

NLC, like SLN, are solid at room and body temperature; however, they have a lower melting point than SLN due to imperfections and their amorphous nature, thus providing more space for dissolution. and correct loading of the drug in its liquid part [18].

Now, for both types of lipids nanocarriers, there are different formulation techniques, which constitute one of the most important parameters when synthesizing them, since these techniques have a fundamental role in the performance and physicochemical characteristics of the nanoparticles [17,18].

Thus, the choice of this technique must be based on parameters such as the physicochemical properties and stability of the drug to be incorporated, the desired characteristics and stability of the lipid nanocarrier, and the availability of the instruments and equipment necessary for production [17,18].

**Figure 5:**
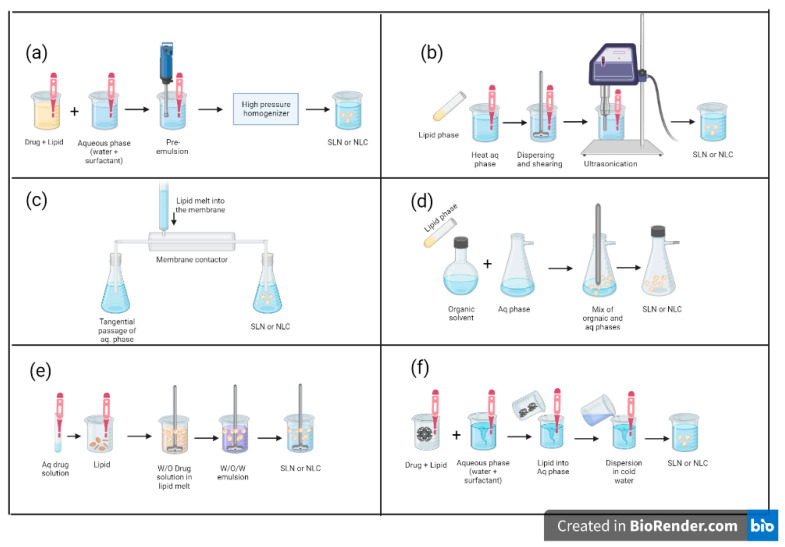
Formulation techniques of SLN and NLC. Type 1 techniques: (**a**) homogenization at high pressure and (**b**) high-speed homogenization. Type 2 techniques: (**c**) emulsification through contact membranes, (**d**) solvent evaporation, (**e**) double emulsion, and (**f**) microemulsion. Adapted from Ganesan and Narayanasamy [23].

Formulation techniques can be divided into type 1, made up of high-energy techniques for the dispersion of the lipid phase, and type 2, made up of techniques where the precipitation of nanoparticles from homogeneous systems is sought. Below is a summary of the main techniques of each indicated type, that is graphically represented in Figure 5 [18,24].

- Type 1 techniques:
  - Homogenization at high pressure: it consists of generating a great shear force, turbulence, and cavitation in the lipid through the application of high amounts of energy. It gives rise to nanoparticles with a low polydispersity index (PDI). The formation of fine particles is favored by increasing pressure, temperature, and homogenization cycles. Hot homogenization is considered emulsification. In cold homogenization, solid particles are ground by applying pressure. However, high pressures can cause particle coalescence [14,17,18,24,56].
  - High-speed homogenization: homogenizers reduce the size of lipid particles in water to a nanometric scale, generating specific centers of high pressure and temperature that give rise to lipid nanoparticles [14,24,56].
- Type 2 techniques:
  - Emulsification through contact membranes: contact membranes have the advantage of providing particle size control through production parameters, and they also constitute a scalable method. They consist of ceramic membranes, with a small diameter and surface area, behaving like parallel capillaries. They contain an active ZrO_2_ layer on an Al_2_O_3_-TiO_2_ support. To formulate, the oily phase is placed in a nitrogen atmosphere with a regulated temperature above its melting point, feeding the module through the pores; at one end, the aqueous phase at a controlled temperature, tangentially inside the membrane, meeting the lipids and generating small droplets. The expelled flow is brought to room temperature by stirring [24,57,64].
  - Solvent evaporation: it consists of dissolving the lipid phase in an organic solvent immiscible with water and then emulsifying this phase with the aqueous one. The precipitation of lipid nanoparticles in aqueous medium occurs during the evaporation of the solvent under reduced pressure [24,57].

A technique derived from the previous one is called double emulsion, which is used to a lesser extent in lipid nanoparticles; however, on some occasions when it is required to incorporate hydrophilic drugs in this type of nanoparticles, its encapsulation is made with a stabilizer to avoid dispersing in the external aqueous phase when the solvent is evaporated [57].

- Microemulsion: this technique seeks to dilute a microemulsion in water. To do this, a microemulsion is first obtained at a temperature 10 or 15 °C higher than the melting temperature of the solid lipid. Then the heat of said microemulsion is dissipated at a temperature between 2 to 4 °C under constant agitation. No higher energy costs are required. In the microemulsion there will be drops of tens or hundreds of nanometers of lipid phase dispersed in the aqueous phase. The microemulsion–water ratio should be no greater than 1:25 or 1:50 [24,27,65].

## 4. New Formulation Methodologies

A common factor in the aforementioned techniques is the application of thermal heating or conductive heating. This also constitutes the traditional form of elaboration of organic synthesis [66]. Generally, formulation ingredients are subjected to conductive heating with an external heat source, either a water or oil bath. This type of heating depends on the thermal conductivity of each material, so the temperature of the mixture is consequently irregular, and the container will always be hotter than its interior. Therefore, the efficiency in terms of energy transfer is quite low, giving rise to products with non-uniform characteristics [67].

### 4.1. Microwave-Assisted Synthesis (MAS)

In order to overcome the aforementioned barrier, Shah et al. in 2014 [27] proposed for the first time the formulation of solid lipid nanoparticles by means of microwave-assisted synthesis based on the fact that this heating is dielectric in nature, that is, it depends on the dielectric properties of materials. In this technique, microwaves couple with particles present in the formulation container, where the temperature rises rapidly [27]. As there is no dependency on the thermal conductivity of the reaction vessel, dipole polarization and/or ionic conductance will occur, by instantaneous overheating. Obtaining in this way, formulations with better characteristics and greater homogeneity [27,68].

#### 4.1.1. The Chemistry Involved

**Figure 6:**
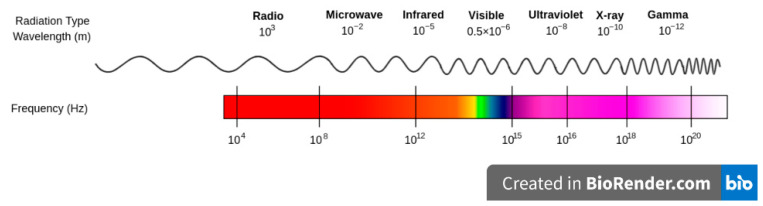
Representation of the electromagnetic spectrum. (Created using Biorender).

The electromagnetic spectrum comprises the classification of all kinds of radiation present in the universe (Figure 6). At the lower end of this spectrum (frequencies between 0.3 GHz and 300 GHz, corresponding to wavelengths from 1 mm to 1 m) is the type of radiation called microwaves. The microwave frequencies authorized in international agreements are used in satellite radio (2.3 GHz) and wireless devices (2.4 to 5 GHz), the latter being the most common. The 2.45 GHz frequency provides an appropriate penetration depth of interaction with chemical reagents, as well as an accessible operational readiness [68,69].

A synthesis microwave has oscillating electric and magnetic fields, emitted perpendicularly at the travel direction of photons and they move at the speed of light (3 × 10^8^ m/s) [68].

Magnetic field interactions do not occur during the chemical synthesis process and the electric field is responsible for heating substances. Therefore, the microwave synthesis chemistry is based on “dielectric heating”. This type of heating refers to the ability of a material to absorb and convert microwave energy into thermal energy. The dielectric properties of a material are responsible for generating its heating in a microwave synthesis. This can occur in two ways: dipolar polarization or heating by friction and ionic conductance [68,70,71].

**Figure 7:**
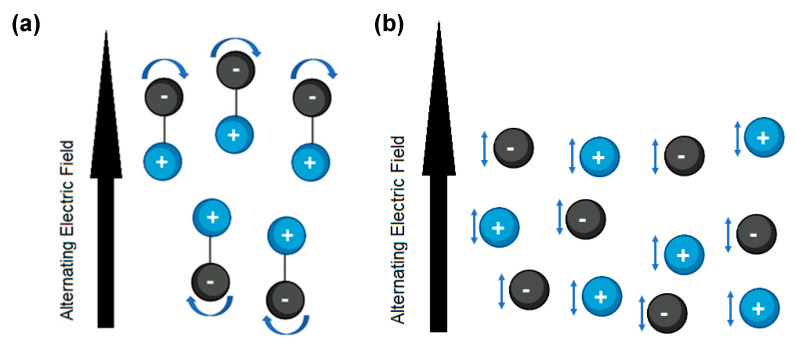
Ways of heating a material by microwaves incidence in microwave-assisted synthesis. (**a**) Graphical representation of the dipolar polarization. (**b**) Graphical representation of Ionic conductance. (Created using Biorender).

In dipole polarization, the dipoles’ ability to orient themselves, as well as their mobility in an alternating electric field determines the heating capacity. This is how this principle is applicable to dipoles or polar molecules, generally in a liquid state, such as water. Frictional heating involves the attempt of these dipoles to align with alternating electric fields, by partial or complete rotation; however, at the common frequency (2.45 GHz), the field changes 2.45 × 10^9^ times per second, which makes this alignment difficult. That is why the molecules in their attempts to align and realign themselves with the electric fields rotate back and forth, giving rise to dielectric friction heating (Figure 7a) [69,70,72].

In ionic conductance, species such as charged ions in solutions or electrons in solids are moved by the alternating electric field. These are aligned with the oscillation of said field (Figure 7b), which generates greater kinetic energy that is transformed into heat through molecular collision [69,70,72].

The dielectric properties of a specific material determine its behavior in dielectric heating. This corresponds in first place to the relative permittivity (*ε*′) of electric charge storage, that is, to the difference in electric capacity of the full or under vacuum capacitor of the synthesis microwave reactor (Equation (1)). In addition, the dielectric loss factor (*ε*″) is also involved, which indicates the amount of microwave irradiated on the sample that is lost when irradiated in the form of heat (Equation (2)) [66,69,71,73].

(1) $$\varepsilon^{\prime }=\frac{C}{C_{0}}$$

(2) $$\varepsilon^{\prime \prime }=\frac{\sigma }{2\pi f}$$

where:

- C corresponds to the electrical capacity of the filled capacitor.
- $C_{0}$ corresponds to the electrical capacity of the capacitor under vacuum.
- σ is the dielectric conductivity.
- f is the microwave frequency.

Both properties establish the conversion of microwaves into heat and are related by a “dissipation factor” tan δ, corresponding to the ratio of the dielectric loss factor and the relative permittivity (Equation (3)) [66,69,71,73].

(3) $$\tan\delta =\frac{\varepsilon^{\prime \prime }}{\varepsilon^{\prime }}$$

For the formulation of SLN, different solvents previously used according to the techniques mentioned in Section 3 have been reported. Currently, SLNs are characterized in that water is the preferred solvent. That is why, when elaborating SLN in microwave synthesis, it will be mainly sought that the formulations follow a specific constitution where water is the solvent, unless the use of another solvent is required to achieve the desired results. In this way, the type of solvent will establish the microwave absorption capacity where low, medium, or high microwave absorbers are found, according to the value corresponding to the dissipation factor [27,72,74]. This classification will depend on the frequency and specific temperature of the microwave reactor, since at least the dielectric loss factor is directly proportional to the temperature. However, the different changes that may occur in the common characteristics of a material, such as deformations, can alter said dielectric loss factor [68]. On the other hand, one of the advantages of using microwave synthesis to formulate SLN is that microwaves have minimal effects on the structure of materials. This is because the energy of microwave photons (~1 J/mol) is less than the energy needed to break chemical bonds such as C-C, C-H, C-O, O-H, and H-H (4–800 kJ/mol) [64,68].

It is important to take into account that this type of synthesis the main obstacle is the difficulty in scaling up because of the low penetration depth of microwaves [75]. To overcome this, with the passing of the years, there has been a proposal based on the combination of microwave chemistry and flow chemistry. For this, some flow microwave systems have been developed in both academic and industrial communities. An example of that is listed in the personal account of Hiromichi Egami and Yoshitaka Hamashima in 2019, summarizing progress of scalable microwave-assisted continuous synthesis using the SAIDA flow microwave apparatus. They demonstrated the scale-up of nine fundamental organic reactions using a novel flow microwave system developed by the academic-industrial alliance between the University of Shizuoka, Advanced Industrial Science and Technology, and SAIDA FDS, demonstrating that this strategy allows the productivity on scalation from grams to kilograms scale [75].

#### 4.1.2. Advantages of Microwave Heating over Conventional Heating

**Figure 8:**
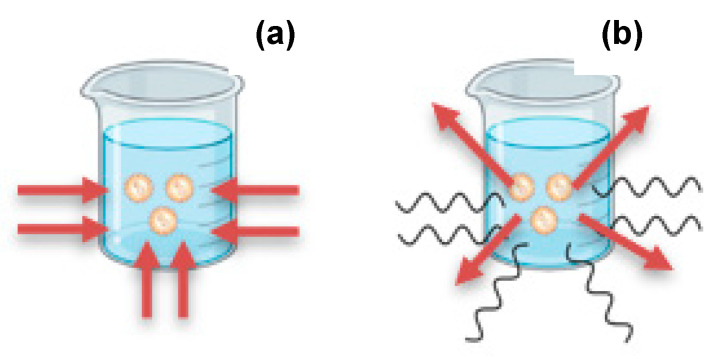
Schematic representation of heating incidence in (**a**) conventional technique and (**b**) microwave-assisted synthesis. (Created using Biorender). Red arrows: heating incidence, wavy lines: microwave energy.

Conventional heating typically requires an external source to provide heat such as controlled temperature bath systems or electric heaters. In this way, the barrier between the reaction vessel and the heat source acts as a collecting medium for thermal energy, which is then exchanged with the reaction solvent. Thus, the reactants involved begin to interact to consequently become the product of the reaction [69]. The detail is that the materials involved do not reach a homogeneous temperature at which the reaction occurs, since heating occurs due to thermal gradients, which causes non-uniform transformations with further uniform production [68,71]. According to the chemistry involved in microwave-assisted synthesis, it offers the possibility of constant heating of the reagents throughout the reaction medium, transferring heat quickly and uniformly, where thermal energy intervenes from the nucleus of the materials involved to the outside, thus generating an efficient form of homogeneous production [69]. However, certain parameters such as microwave field intensity, volume of the material, reaction time, dielectric properties of the material, and geometry of the reaction vessel are what will establish the dimension of the heating. On the other hand, the materials can absorb the energy emitted by microwaves, culminating through dipolar polarization or ionic conduction in electronic relaxation translated into rapid heating of the reaction [68,71]. Additionally, in Figure 8, there is a representation of the comparison of the heating incidence in conventional and MAS techniques.

The microwave heating rate is a fundamental parameter of the MAS, since, in each period of electromagnetic energy, the microwave can transfer energy in 10^−9^ s and relax kinetically at the molecular level in 10^−5^ s. Thus, the relaxation corresponding to the transfer of energy will relax at a faster rate than that of the molecules, implying an imbalance and instantaneous temperature that are decisive in the kinetics of the system. This can translate into higher reaction rates and better product yields. However, microwaves are not able to affect the activation energy or collision energy between the particles of the materials subjected to the microwave synthesis reaction [27,69].

According to what was described above, heating by MAS can increase the reaction rate between 10 and 1000 times more than conventional heating. This is how, for example, reactions that under normal conditions occur in a period of 10 h, could take 35 min in microwave heating [72].

#### 4.1.3. SLN Formulated by Microwave-Assisted Synthesis

Although the chemical principle and the significant difference that exists between conventional heating and heating by microwave-assisted synthesis have shown promising results in terms of chemical reactions, this technique was not applied for the elaboration of SLN until 2014, where Shah et al., based on the theoretical principles exposed previously, were able to demonstrate that SLN formulations normally elaborated by the microemulsion technique with sizes from 360 to 600 nm and polydispersity indices (PDI) of 0.300 can be scaled to a “single pot” by means of MAS to obtain corresponding sizes between 200 to 250 nm and PDI of 0.150. These results reflected that the thermal energy involved from the particle nuclei of the excipients in SLN formulations can translate into greater homogeneity and monodispersion, as well as smaller sizes and better stabilities. However, they do not interfere with the potential difference of these colloidal carriers since the values corresponding to the zeta potential (−20 mV) did not show significant changes. In this way and from this research, new works have been oriented towards obtaining improved SLN formulations, with better properties in terms of encapsulation of various drugs, constituting a new aspect in the preparation of this type of nanocarriers [27].

By 2016, the same authors, Shah et al. [76], focused on another point of view regarding SLNs produced by microwave-assisted synthesis. They now directed their efforts to verify the encapsulation efficacy and release profile of lipophilic drugs. This is how they studied the encapsulation of clotrimazole, a synthetic imidazole-type antifungal drug that has shown anticancer and antirheumatoid activity. However, this drug exhibits poor and very low solubility in water when administered orally, which compromises its bioavailability. To overcome this barrier, they incorporated clotrimazole into SLN stearic acid using Tween^®®^ 20 as the surfactant and water as the solvent. The synthesis was in “single pot” by MAS. The physicochemical characteristics obtained corresponded to a size of 274 ± 2 nm with a PDI of 0.180 and a zeta potential of −21 ± 2 mV. Regarding the release profile, a gradual but incomplete release of the drug was observed after 24 h incubation with phosphate-buffered saline (pH 7.4) with anomalous non-Fickian behavior. Furthermore, it was found that the fungistatic activity of the drug was maintained when it was incorporated into SLNs. Regarding cell viability, all concentrations of SLN used by the authors were not shown to be toxic. It was also concluded that through endocytosis the designed SLNs are internalized in the A549 epithelial cell lines. Thus, according to the results obtained in this study, the SLNs synthesized in microwave synthesis are good carriers of clotrimazole, facilitating its administration by different routes [76].

On the other hand, the efficacy of encapsulating non-steroidal anti-inflammatory drugs (NSAID), such as indomethacin, ketoprofen, and nimesulide, in MAS-made stearic acid with Tween^®®^ 20 as a surfactant and water as a solvent has been shown to be effective. Regarding the physicochemical characterization, they obtained sizes in the range of 250–300 nm and PDI ˂ 0.260 and a zeta potential value of ~20 mV, for the three encapsulated drugs. Regarding the encapsulation efficiency, they obtained a value of ~70–90%, being considered as high. They had a load capacity of 3.6–4.6% (*w*/*w*). The good level of these two values reflects that the MAS of “single pot” favors that the encapsulation of the drug occurs simultaneously with the formation of the nanovehicles. Referring in this way to the fact that there is a greater effective surface during the SLN synthesis process, for which the drug does not necessarily penetrate the cover to be absorbed. The release profile initially corresponded to a “burst” release, followed by a “sustained” release. The drug molecules that were trapped on the surface of the SLNs were released first, followed by the release of molecules that diffuse from the nucleus. Considering cytotoxicity, it depended on the concentration. Finally, SLNs were shown to be taken up by epithelial cells and the authors concluded that these NSAID drug nanocarriers will facilitate their administration by topical, oral, and/or nasal routes [77].

Ionic lipophilic drugs such as miconazole nitrate and econazole nitrate can also be efficiently encapsulated in SLN. These drugs according to the biopharmaceutical classification system are classified as type II and IV, respectively, so lipid-based formulations are suitable for them. According to this, SLN of stearic acid, Tween^®®^ 20, and water with sizes between 250 to 300 nm, PDI < 0.200 and zeta potential values of 20 ± 1 mV for encapsulated miconazole nitrate and 12 ± 4 mV for encapsulated econazole nitrate. Regarding the encapsulation efficiency, this has been located within a range from 72 to 87% and a load capacity range from 3.6 to 4.3%, which reaffirms that MAS favors the encapsulation of lipophilic drugs in SLN. The release profiles of this type of drug have corresponded to an initial burst release followed by a slow and sustained release up to 24 h. In addition, this type of nanocarrier has been proven to be non-toxic at concentrations below 100 μg/mL [65].

Innovating even more in terms of SLN by MAS formulations, the first study of SLN by MAS was carried out using the phase inversion temperature technique. This methodology allows the elaboration of nanoparticles with sizes smaller than 100 nm, starting from the fact that, when the temperature varies, the inversion of an O/W emulsion to a W/O emulsion is generated, reaching the midpoint where the surfactant is affine to both phases. The researchers elaborated a formulation composed of Brij 78, vitamin E TPGS, and lipid nuclei based on triglycerides (such as trilaurin, trimyristin, or migliol 812). These nanocarriers were reported by previous methodologies to be ~200 nm in size and they are able to target tumors, deliver chemotherapeutic drugs in vivo, and overcome multidrug resistance. MAS managed to reduce the reported sizes until obtaining lipid nanoparticles of sizes less than 30 nm in one minute of exposure, with PDI ˂ 0.300 and zeta potential between −3 to −5 mV. These new physicochemical characteristics of the nanocarriers were obtained with 1 min of exposure to microwaves. In this way, they corroborated the viability of using MAS for the formulation of lipid nanoparticles by phase inversion temperature, being also physically stable and optically transparent. Improvements that are not shown to be possible using conventional thermal heating [78].

Continuing in terms of innovation regarding the introduction of MAS as a formulation technique for lipid nanoparticles, NLC were developed for the first time in 2018. This is with the aim of observing if there were variations in the properties reported by the hot ultrasonication formulation methodology. The designed NLCs were directed towards the encapsulation of the antiretroviral agent zidovudine (AZT) since, although it is a type I drug according to the biopharmaceutical classification system, it has low oral bioavailability and the possibility of producing granulocytopenia and anemia. The development and optimization of NLC formulations were supported by a quality-by-design (QbD) approach. The formulation components corresponded to Precirol^®®^ ATO 5 as lipid solid, Miglyol^®®^ 812 as lipid liquid, Tween^®®^ 80 as surfactant, water, and AZT. Regarding the physicochemical characteristics obtained, in terms of hot ultrasonication, sizes of 266 ± 4 nm, PDI of 0.168 and a zeta potential of −29 ± 2 mV were achieved. In contrast, for this same formulation made by MAS, the results were 113 ± 3 nm in size, a PDI of 0.216, and a zeta potential of −20 ± 1 mV. As it could be observed, MAS provided smaller sizes. According to these physicochemical characteristics, both formulations presented an adequate profile for oral administration (particle size between 100 and 300 nm, PDI < 0.3 and negative zeta potential > −20 mV). Similarly, both formulations were physically stable for at least 45 days and were not toxic to Jurkat T cells. They also exhibited spherical shape and release studies presented controlled release of AZT under gastric and plasma-simulated conditions. Therefore, this research showed that it is possible to make NLC by the MAS method [79].

Therefore, with the aim of expanding the fields of application of SLN prepared by MAS, observing that their formulation is possible and that they exhibit better physicochemical characteristics, the authors Aldawsari, H and Singh, S in the year 2020 developed research aimed at improving the biocompatibility of available cancer therapies. This is how they encapsulated cisplatin, one of the most powerful chemotherapy drugs prescribed for the treatment of most solid tumors, in SLN of stearic acid (solid lipid), Tween^®®^ 80 or Solutol HS 15 as surfactants and water. The formulation method was “single pot” MAS, to obtain spherical nanovehicles of 75 nm, with a PDI of 0.311 and a zeta potential of −20 mV. Cisplatin encapsulation showed an efficiency of 71.85% and a hemolysis study found that the formulation was safe in blood. In vitro release was found to be 80% over 24 h. The IC_50_ of the SLNs had a value of 6.51 ± 0.39 μg/mL and their cytotoxicity were tested in the MCF-7 breast cancer cell line. Thus, this study allowed us to conclude that the administration of cisplatin encapsulated in SLN formulated in microwave synthesis can provide greater sustainability in breast cancer therapy with superior biocompatibility to previous therapies [50].

Following the same line of research focused on particle size reduction, the effect of MAS on SLN formulation, Sharma et al. in 2021 elaborated SLN loaded with luliconazole using stearic acid as lipid solid and Pluronic F-68 as surfactant with water. Surfactant concentration was also an objective parameter in this study. Among the defined findings of this work, they officially reported what can be evidenced in previous works, corresponding to microwave power having a greater influence on particle size than on zeta potential. However, for this specific investigation, microwave power and Pluronic F-68 concentration did not exhibit a significant influence on luliconazole entrapment. Physicochemical characterization values corresponding to 91 nm in size, 0.256 PDI and a zeta potential of −20 mV were reported. The trapping efficiency of luliconazole corresponded to 96.68% and a total release (100%) of luliconazole was obtained in 24 h at pH 7.4 following the Higuchi square root kinetics, with diffusion through the matrix being the main factor. release mechanism. The solid lipid nanoparticles exhibited excellent antifungal action with a minimum inhibitory concentration of 6.25 µg/mL against *Candida albicans* (MTCC 227) and 12.5 µg/mL against *Aspergillus niger* (MTCC 8189). That is, the use of microwave technology in the design of SLNs of luliconazole is efficient and provides promising results. However, the group recommends evaluating the clinical utility of designed SLNs through in vivo oral and topical administration studies [80].

Finally, regarding the current investigations in which MAS is used for the formulation of SLN, the development of transdermal administration therapies is found. In relation to this topic, Jagdale et al. in 2022 designed solid lipid nanocarriers to encapsulate ketoprofen (KP). KP is limited in its common form of administration since it causes gastric irritation due to its acidic nature, for which its transdermal administration is attractive since it offers the possibility of overcoming this problem. KP SLNs were made using stearic acid as solid lipid, Tween^®®^ 80 as surfactant, and water as solvent. The physicochemical characteristics of the nanocarriers corresponded to a size of 683 nm, a PDI of 0.685, and a zeta potential of −29 mV. While these features are not ideal they constitute the first step in the development of this type of colloidal dispersions in KP transdermal administration. As for the elaborated assays, a trapping efficiency of 74.8% was obtained and a forty-fold improvement in drug solubility was also evidenced. The release was burst at first, followed by a controlled release for 8 h in vitro drug release. Therefore, the KP SLNs broadly showed, at least for a first development, promising behaviors, and characteristics as an alternative therapy for transdermal administration [51].

**Figure 9:**
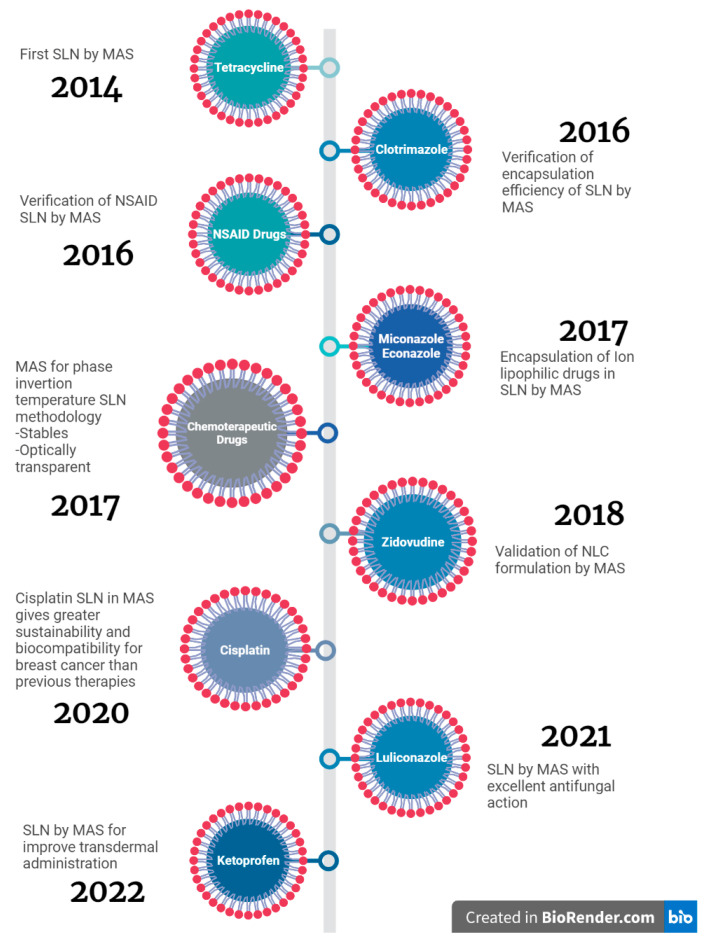
Timeline of investigations of solid lipid nanoparticles and nanostructured lipid carriers using microwave-assisted synthesis. [27,50,51,65,76,77,78,79,80].

As is evident and exposed in Figure 9 timeline, at present, there are not many investigations that refer to the use of MAS for the formulation of SLN or NLC, since it is a novel technique in this area. Work has been done to achieve the standardization of adequate conditions for each of the therapies drug encapsulations and required uses [36,37,38,39,40,41,42,43,44,45,46,47,48,49]. For example, encapsulating immunosuppressors such as tacrolimus, improving its ability to penetrate skin layers [40], or even exhibiting a great potency for oral delivery of peptide/protein drugs such as insulin [37]. However, broadly speaking, each of the listed MAS SLN and NLC studies refers to the fact that it constitutes an effective, fast technique that provides smaller sizes and lower PDI. Showing that it allows the encapsulation of different drugs, both lipophilic and hydrophilic, in this type of nanocarriers in a successful way.

### 4.2. Ultrasound-Assisted Synthesis (UAS)

In relation to the novel techniques for the elaboration of SLN, in addition to the MAS, the UAS stands out. This technique is based on the emission of acoustic waves with frequencies at different intensities that are characterized by exceeding the human hearing range (>16 kHz) [81]. These intensities can be classified as low and high, being inversely proportional to the frequency. Low-intensity ultrasound has frequencies between 1 and 10 MHz with very low powers (<1 W/cm^2^), so they do not cause physicochemical changes that involve destruction. This type of ultrasound is used both in detection and diagnosis in the environment. On the other hand, high-intensity ultrasound has frequencies between 16 to 100 kHz and powers between 10 to 1000 W/cm^2^. It is commonly used to alter the properties of materials, favoring their physical transformation, such as emulsion manufacturing, depolymerization, deflocculation, and particle size reduction [81,82,83].

#### 4.2.1. The Chemistry Involved

Acoustic energy is not directly absorbable by molecules so its conversion into chemical energy that facilitates this absorption is called acoustic cavitation. This phenomenon is based on the transfer of acoustic waves through a liquid that has pressure waves in the form of expansion waves that break the cohesion of liquid media, generating microcavities and compression waves that compress the generated microcavities. This process causes the formation of bubbles that oscillate at a speed of 20,000 times per second and grow by tens of μm. These bubbles, when found at low ultrasound intensity, only oscillate in the same way, so they are not prone to implosion [81,82]. On the other hand, at high ultrasound intensities the oscillation is no longer stable, it changes in different directions and the bubbles begin to implode, rapidly releasing their internal energy. Consequently, the main mechanism of action of the sonochemical effect occurs, where these implosions or bubble shocks known as “transient cavitation” originate free radicals, shock waves, very high localized temperatures, and high pressures [84,85].

The particle size distribution can be classified as a physical effect of ultrasound caused by transient cavitation. However, the driving forces that specifically generate nanoparticles are still under study. Sonication has been found to be capable of breaking down particle aggregates, reducing sizes to nanometric scales, either alone or in conjunction with other nanoparticle formulation techniques. The characteristic random Brownian motion of colloidal suspensions tends to favor particle aggregation due to the intervention of attractive surface forces [81,82,84]. Changes in pressure, temperature, or volume can favor the rearrangement of particles in such a way that they self-assemble avoiding aggregation. Transient cavitation heating occurs throughout the entire ultrasound frequency range, while physical processes such as emulsification and nanoparticle generation generally occur at low frequencies [82].

In addition, it is necessary to take into account that the sonication process is highly dependent on the configuration of the equipment used. These generally must be composed of an electrical generator with a certain nominal power, a simple/multiple piezoelectric transducer that vibrates at ultrasonic frequencies, transforming electrical energy into sound waves, and a titanium emitter that transmits sonication waves to the environment. The most used equipment such as ultrasonic horns and ultrasonic cleaning baths operate at frequencies between 20 and 40 kHz respectively, however, the latter do not provide adjustable powers and complexity in terms of bath temperature control, which does not favor the generation of nanoparticles. Therefore, the probes in common use that do allow the selection and control of these parameters have been found to be the ones indicated for the formulation of nanocarriers [81,82,83,84,85].

However, conventional ultrasonic liquid processing technology has been limited in time for scaling up processes, restricting its industrial implementation [86]. Few researchers are focused on this limited industrial scalation, such as the work of Peshkovsky et al. in 2013. They managed to produce translucent oil-in-water nanoemulsions, optimizing it on a laboratory scale and then directly scaling up with the use of barbell horn ultrasonic technology (BHUT) without reducing the ultrasonic amplitude. Additionally, they demonstrated that, given sufficient generator and transducer power, it is possible to construct BHUT-based ultrasonic liquid processors utilizing half-wave barbell horns with output diameters up to 75 mm, further process scale-up by a factor of about five is possible (pilot to industrial scale) [87].

#### 4.2.2. SLN Formulated by Ultrasound-Assisted Synthesis

Since a high amount of energy is necessary to generate nanoscale particles, different types of SLN have been formulated using ultrasonication methods alone or in combination with other nanoparticle manufacturing methods. One of the main characteristics that have governed the use of ultrasonication for SLN has been the low particle size it provides, which is in the range between 30 to 180 nm, as well as the low shear stress. However, it is necessary to clarify that a risk of using this method is associated with contamination by metals derived from the degradation of the ultrasound probe, which can lead to the oxidative degradation of the bioactive molecules involved in the formulation. This obstacle has been overcome by coating said probe [24].

This is how various investigations have been carried out under this methodology to evaluate the ability to improve characteristics previously obtained by other means and to directly manufacture SLN. For this, it was found that SLN with incorporated safranal polyphenolic components synthesized by ultrasonication showed smaller sizes (112 nm) compared to its original methodology by high-pressure homogenization (233 nm) [88].

On the other hand, high-energy-based ultrasonic emulsification–evaporation has shown efficiency in the preparation of SLN. For this, a study in which the lipid solid, organic solvent, emulsifier, and bioactive materials were mixed to obtain an emulsion, and ultrasonicated at temperatures above the melting point of the solid lipid used for a set time. Like homogenization/ultrasonication, SLNs are formed by dispersing the emulsion in cold water with surfactant after evaporation of the solvent. This process is directly associated with transient cavitation forces in the dispersion [89].

Although few studies have been carried out with the use of ultrasound, it is evident that it presents promising results in terms of improving the physicochemical characteristics obtained through other SLN production methodologies. In the same way, some research has been carried out, aimed at innovation in the formulation of NLC where the energy provided by ultrasound leads to the reduction of the particle size of the lipid emulsion of the core. This, by breaking the lipid mixture into small droplets, leads to improvements in the solubility of the oil in the solid. These fine oily compartments precipitate out of the solid lipid matrix during the cooling period. Increasing solid unsaturated lipids has been shown to favor cases where the encapsulated molecules are prone to attack by radicals generated in lipid oxidation reactions, providing them with greater protection. Thus, probe ultrasonication produces NLC with a better PDI than those made through other techniques such as high-pressure homogenization. The values 0.100 and 0.250, providing monodispersion, favor the long-term stability of these nanocarriers [90].

Regarding the preparation of SLN where the encapsulated molecule is arranged in the outer shell of the same, an investigation was carried out using shea butter as a solid lipid and curcumin as an encapsulated agent. The encapsulation efficiency of SLNs with larger diameters is lower than those with smaller diameters, having a higher surface area to volume. Studies carried out by X-ray diffraction (XRD) were able to confirm the arrangement in the outer cover of the SLN of curcumin. Therefore, the polar groups of curcumin interacted with and the hydrophilic part of the surfactant, favoring the “burst” release of curcumin [91].

Optimizing processing for sonication is essential to achieve SLNs with ideal physicochemical characteristics (smaller sizes, polydispersity indices, and high zeta potential). This was demonstrated by incorporating Ficus religiosa L extract into SLN. Again, using the combination of hot homogenization and ultrasonication. This study provided results that corresponded to the fact that both the increase in exposure time and the intensity of sonication influence the obtaining of smaller particle sizes. Thus, a homogenization speed of 15,000 rpm, a homogenization time of 30 min, a sonication amplitude of 50%, and a sonication time of 5 min showed high stability during storage for 6 months. In addition, a sustained release profile in ultrasonically formulated SLNs was found to be influenced by the large surface area and high diffusion coefficient resulting from the small or low molecular size and viscosity of the matrix [92].

Likewise, the high-speed ultrasonication technique at high temperatures has also proven to be efficient in providing greater stability and smaller sizes in the formulation of SLNs. This is done by heating the lipid solid 5 or 10 °C above its melting temperature and its subsequent dispersion in the aqueous phase with surfactant at the same temperature, applying homogenization and sonication that stimulates the reduction in the size of the emulsion droplets. The SLNs are then dispersed by gradual cooling [93].

Dolatabadi et al. [94] elaborated NLC for curcuminoids encapsulation with solid lipid glyceryl palmitostearate and MCT oil as medium-chainin triglyceride, along with surfactant Poloxamer 188. The average particle size for the NLCs was 152 nm. Furthermore, they obtained zeta potential values of −20 mV corresponding to high physical stability. Regarding the encapsulation efficiency, this corresponded to 97%. Additionally, in vitro release studies were carried out that revealed that the NLC of curcuminoids presented a prolonged release over time. Pharmacokinetic studies confirmed that these lipid nanoparticles could potentially improve the maximum plasma concentration (Cmax) and the area under the concentration–time curve (AUC) of curcuminoids compared to the pure drug, thus representing novel delivery systems for the formulation of drugs characterized by insufficient oral bioavailability [94].

**Figure 10:**
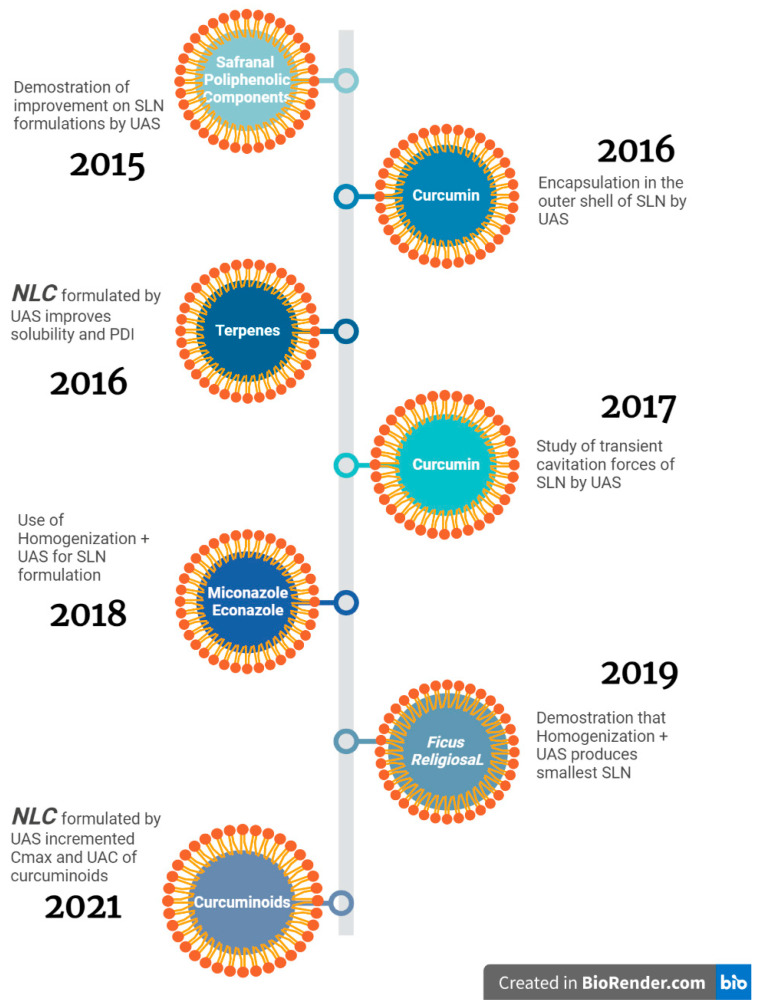
Timeline of investigations of solid lipid nanoparticles and nanostructured lipid carriers using ultrasound-assisted synthesis [88,89,90,91,92,93,94].

As summarized in Figure 10, ultrasound and combinations with other high-energy synthesis methods have provided favorable results in the production of stable SLN and NLC at experimental laboratory scale. Its operational simplicity and easy availability, in addition of its green approach of high energy efficiency, makes ultrasound a promising method in future industrial scalation on this type of nanocarriers formulations.

## 5. Green Chemistry behind MAS and UAS

According to the characteristics described in the MAS and UAS, both methodologies contribute to the definition of the concept known as “green chemistry”, defined as a new approach to the design of chemical products and processes, reducing or eliminating the generation of harmful products [85,95]. Thus, by coupling these techniques as new formulation strategies for SLN, it is possible to establish two new methodologies focused on the principle of green chemistry, converging to the point of not using organic solvents to obtain lipid nanoparticles, using water instead. On the other hand, as mentioned above, the use of physiological lipids for the preparation of formulations of this type also constitutes a point in favor in terms of the biodegradability of nanoparticles [96,97,98,99,100,101].

In addition, MAS and UAS favor SLN and NLC formulations due to greater energy efficiency and, in the particular case of MAS, saving time. This is evidenced in the results obtained from the first SLN formulated by MAS in 2014 by Shah et al., where the efficiency of the thermal energy involved from the particle nuclei of the excipients in the formulations can be translated into greater homogeneity and monodispersion, as well as smaller sizes and stabilities obtained in less time than that used in the traditional microemulsion technique [27]. In the UAS, it can also be evidenced in the work of Khameneh et al., where the higher energy efficiency of this new SLN formulation methodology was shown to generate smaller sizes (112 nm) of nanocarriers with incorporated safranal polyphenolic components compared to their original methodology by high-pressure homogenization (233 nm) [88].

In summary, both types of synthesis provide better product yields and by controlling experimental parameters such as temperature gradients, frequency, irradiation at different wavelengths, power, and pressure, they have an influence that recent research has shown to generate better particle sizes, homogeneity and morphologies than conventional formulation methods [35,102,103,104]. Additionally, a schematic representation of the advantages and disadvantages of this methodologies over the conventional ones for lipid nanocarriers formulations can be observed in Table 2. In this way, MAS and UAS can be considered as a valuable contribution to the nanoworld, providing the possibility of a greener manufacturing approach in the near future.

## 6. Limitations and Future Challenges

This review showed the potential of MAS and UAS for the production of SLNs, representing a methodology that is currently receiving increasing interest. The literature evidence clearly shows improvements of physicochemical parameters, such as size and PDI, as well as interesting features in terms of encapsulation and release profiles. However, an expansion of this research field is necessary to fully establish all the advantages these techniques can bring for the preparation of lipid nanoparticles [24,40,88,105].

Interestingly, the use of UAS for the formulation of lipid nanoparticles without combining with any other technique is still elusive. Many current challenges apparent in the production of SLN might be overcome by MAS, UAS or combinations of it, and the increasing number of studies show the efforts in this area [66,106,107,108,109,110,111,112,113,114].

Once the aforementioned limitations are overcome, it will be evident that the next future challenge will be focused on the scalability of both methodologies for SLN and NLC formulations, with industrialization perspectives. However, based on the growing needs of the world community, related to drug discovery against emerging diseases as COVID-19, or existing ones such as cancer or diabetes, it is clear that teamwork between interdisciplinary health researchers can lead the generation of technologies such as pandemic vaccines [21,22,115]. Therefore, it is no longer a dream to think about the scalability of MAS and UAS for lipid nanoparticles, using, for example, a flow microwave reactor as SAIDA operated by Hiromichi Egami and Yoshitaka Hamashima in 2019 [75]. In a combined microwave and ultrasound reactor, Pawełczyk A, et al. in 2018 [116] demonstrated that the implementation of this technology is more than just imagination, so it could be adapted in the future for the production of SLN and NLC.

## 7. Conclusions

This review was focused on providing a description of the basic principles of MAS and UAS that make them new strategies with a green approach to formulation in the field of lipid nanostructures. Both syntheses constitute simple, fast, safe, and profitable technologies that, in terms of scaling up, offer promising opportunities for the development and production of stable, monodisperse, and small-sized nanosystems, as has been seen in the different investigations carried out to date. The MAS offers the possibility of influencing a formulation in a localized manner, causing thermal energy to be involved from the interior of the nucleus of the particles that make up the excipients of the formulation, to the exterior, favoring the formation of nanostructures, managing to avoid the coalescence of particles. For its part, high-intensity ultrasound irradiation provides unique formulation conditions through acoustic cavitations. The use of ultrasound, by itself or in combination with other encapsulation approaches, is capable of rapidly and efficiently generate lipid nanocarriers. The action chemistry of MAS and UAS were discussed, as well as the necessary parameters that have been used in the formation of SLN together with what the advances that each of the results obtained in different research works have meant in terms of stability, particle size, and process effects on functionalities. Finally, these types of nanoparticles formulated using these techniques have been shown to have a positive influence on the functional properties of the different encapsulated molecules, such as solubility, dissolution, availability, and release profile. However, since these are such new procedures, it is necessary to continue conducting research to support the results obtained so far.

## Figures and Tables

**Table 1:** Drugs incorporated into SLN in recent years with promising results.

Drug	Lipid Matrix	Size (nm)	Polydispersity Index	Reference
Docetaxel	Glyceryl dibehenate	128 ± 2	0.153	Oliveira, M. et al. (2020) [36]
Insulin	Glycerol tripalmitate	171 ± 2	0.230	Yining, X. et al. (2018) [37]
Ethambutol	Glyceryl dibehenate	60 ± 4	0.253	Nemati, E. et al. (2019) [38]
Cyclosporine	Hydrogenated palm kernel glycerides	218 ± 2	0.198	Essaghraoui, A. et al. (2019) [39]
Tacrolimus	Cocoglyceride	153 ± 4	0.250	Kang, J. et al. (2019) [40]
Olmesartan Medoximil	Glyceryl monostearate	152 ± 3	0.290	Pandya, N. et al. (2018) [41]
Curcumin	Stearic Acid	40 ± 10	Only TEM characterization	Wang, W. et al. (2018) [42]
Atorvastatin	Glyceryl dibehenate	256 ± 10	0.260	Yadav, M. et al. (2020) [43]
Lurasidone	Glyceryl monostearate	140 ± 8	0.118	Patel, M. et al. (2019) [44]
Acyclovir	Glyceryl palmitostearate	124 ± 6	0.220	Hassan, H. et al. (2020) [45]
Simvastatin	Palmityl alcohol	129 ± 6	0.220	Syed Z, H. et al. (2019) [46]
Domperidone	Glycerol Triester of stearic acid (best of 5 formulations)	201 ± 6	0.07	Shazly, G. et al. (2018) [47]
Niclosamide	Stearic Acid	204 ± 3	0.328	Maqsood U, R. et al. (2018) [48]
Natamycin	Glyceryl distearate	~84	0.224	Khames, A. et al. (2019) [49]
Cisplatin	Stearic Acid	~75	0.311	Aldawsari, H and Singh, S. (2020) [50]
Ketoprofen	Stearic Acid	~683	0.685	Jagdale, S. et al. (2022) [51]
Fluconazole	Glyceryl palmitostearate (best of 8 formulations)	292 ± 1	0.228	Kraisit, P. et al. (2021) [52]
Streptomycin Sulphate	Glyceryl palmitostearate	218 ± 15	0.240	Singh, M. et al. (2021) [53]
N-acetylcysteine	Glycerol Monostearate	159 ± 15	0.168	Madupoju, B. et al. (2022) [54]
Miconazole nitrate	Semi-synthetic glycerides (best of 5 formulations)	244 ± 27	0.221	Al-Maghrabi, P. et al. (2020) [55]

**Table 2:** Advantages and disadvantages of SLN and NLC formulation techniques.

Formulation Technique	Advantages	Disadvantages
Classics (Type I and II)	✓ Most known and used methodologies for SLN and NLC formulation. ✓ Easy handling. ✓ Industrial scalability.	✓ Generally, multistep processes. ✓ Bigger particle sizes. ✓ Polydisperse size distribution (depending on heterogenous heating)
MAS	✓ Faster formulation technique. ✓ Homogeneous heating (temperature control on microwave reactor, higher energy efficiency) ✓ Smaller particle sizes. ✓ Less polydisperse distributions.	✓ Infrequently used and less known methodologies for SLN and NLC formulation. ✓ Because of its novelty as SLN and NLC formulation technique, it has not been industrially scaled yet.
UAS	✓ Excellent particle aggregation avoiding ability. ✓ Smaller particle sizes. ✓ Higher energy efficiency	✓ Because of its novelty as SLN and NLC formulation technique, it has not been industrially scaled yet. ✓ Highly dependent on the configuration of the used equipment.

## Data Availability

Not applicable.
